# Triaqua-1κ^3^ *O*-μ-cyanido-1:2κ^2^ *N*:*C*-penta­cyanido-2κ^5^ *C*-tetra­kis­(dimethyl­formamide-1κ*O*)-1-holmium(III)-2-iron(III) monohydrate

**DOI:** 10.1107/S160053681102695X

**Published:** 2011-07-16

**Authors:** Hong-Fang Li, Qi-Hua Zhao, Ming-Jin Xie, Fan Yang

**Affiliations:** a Yunnan University, Department of Chemistry, Key Laboratory of Medicinal Chemistry for Natural Resource, Ministry of Education, Kunming 650091, People’s Republic of China.

## Abstract

In the bimetallic cyanide-bridged title complex, [Fe_0.98_HoRu_0.02_(CN)_6_(C_3_H_7_NO)_4_(H_2_O)_3_]·H_2_O, the Ho^III^ ion is in a slightly distorted square-anti­prismatic arrangement formed by seven O atoms from four dimethyl­formamide (DMF) mol­ecules and three water mol­ecules, and one N atom from a bridging cyanide group connected with the Fe^III^ atom which is octa­hedrally coordinated by six cyanide groups. In the crystal, mol­ecules are held together through O—H⋯N and O—H⋯O hydrogen-bonding inter­actions to form a three-dimensional framework. Elemental analysis of one of the precursors and the crystal shows that there is a slight contamination of Fe by Ru. The Fe site displays, therefore, small substitutional disorder with site-occupancy factors Fe/Ru = 0.98:0.02. The two methyl groups of two dimethyl­formamide ligands are positionally disordered with site-occupancy factors of 0.44 (3):0.56 (3) and 0.44 (3):0.56 (3).

## Related literature

For similar complexes [*Ln*Fe(CN)_6_(DMF)_4_(H_2_O)_3_]·H_2_O (*Ln* = La, Ce, Nd, Gd, Pr and Eu), see: Kautz *et al.* (2000 ▶); Mullica *et al.* (2000 ▶); Li, Akitsu *et al.* (2003 ▶); Li, Guo *et al.* (2003 ▶). For *Ln* = Sm and Pr with four coordinating water mol­ecules in the complex, see: Kou *et al.* (1998 ▶); Dai *et al.* (2004 ▶). 
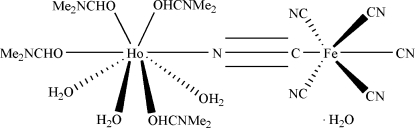


## Experimental

### 

#### Crystal data

[Fe_0.98_HoRu_0.02_(CN)_6_(C_3_H_7_NO)_4_(H_2_O)_3_]·H_2_O
*M* *_r_* = 742.25
Monoclinic, 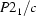

*a* = 17.6587 (15) Å
*b* = 8.9235 (8) Å
*c* = 25.2750 (16) Å
β = 128.208 (4)°
*V* = 3129.5 (4) Å^3^
*Z* = 4
Mo *K*α radiation
μ = 3.03 mm^−1^
*T* = 293 K
0.30 × 0.25 × 0.20 mm

#### Data collection

Bruker APEXII CCD area-detector diffractometer
Absorption correction: multi-scan (*SADABS*; Sheldrick, 1996 ▶) *T*_min_ = 0.464, *T*_max_ = 0.583
19042 measured reflections
6392 independent reflections
5217 reflections with *I* > 2σ(*I*)
*R*_int_ = 0.030

#### Refinement

*R*[*F* ^2^ > 2σ(*F* ^2^)] = 0.042
*wR*(*F* ^2^) = 0.131
*S* = 1.04
6392 reflections
393 parameters
120 restraints
H-atom parameters constrained
Δρ_max_ = 1.95 e Å^−3^
Δρ_min_ = −1.04 e Å^−3^

### 

Data collection: *APEX2* (Bruker, 2007 ▶); cell refinement: *SAINT* (Bruker, 2007 ▶); data reduction: *SAINT*; program(s) used to solve structure: *SHELXS97* (Sheldrick, 2008 ▶); program(s) used to refine structure: *SHELXL97* (Sheldrick, 2008 ▶); molecular graphics: *SHELXTL* (Sheldrick, 2008 ▶); software used to prepare material for publication: *SHELXTL*.

## Supplementary Material

Crystal structure: contains datablock(s) I, global. DOI: 10.1107/S160053681102695X/vn2013sup1.cif

Structure factors: contains datablock(s) I. DOI: 10.1107/S160053681102695X/vn2013Isup2.hkl

Additional supplementary materials:  crystallographic information; 3D view; checkCIF report

## Figures and Tables

**Table 1:** Selected bond lengths (Å)

Ho1—O5	2.412 (4)
Ho1—O6	2.433 (4)
Ho1—O8	2.457 (4)
Ho1—O7	2.461 (4)
Ho1—O4*W*	2.480 (4)
Ho1—O2*W*	2.486 (4)
Ho1—O3*W*	2.489 (4)
Ho1—N6	2.572 (5)
Fe1—C5	1.929 (5)
Fe1—C3	1.935 (6)
Fe1—C6	1.940 (6)
Fe1—C2	1.941 (6)
Fe1—C1	1.944 (5)
Fe1—C4	1.951 (6)

**Table 2:** Hydrogen-bond geometry (Å, °)

*D*—H⋯*A*	*D*—H	H⋯*A*	*D*⋯*A*	*D*—H⋯*A*
O1*W*—H1*WA*⋯N2^i^	0.86	2.05	2.901 (7)	173
O1*W*—H1*WB*⋯N3^ii^	0.86	1.98	2.821 (7)	165
O2*W*—H2*WA*⋯O1*W*^iii^	0.86	1.82	2.660 (6)	167
O2*W*—H2*WB*⋯N1^iv^	0.86	2.06	2.875 (7)	159
O3*W*—H3*WA*⋯N4^iii^	0.86	2.02	2.795 (6)	149
O3*W*—H3*WB*⋯N1^iv^	0.86	1.99	2.839 (6)	168
O4*W*—H4*WB*⋯N4^iii^	0.86	2.13	2.931 (7)	155

Symmetry codes: (i) 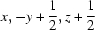; (ii) 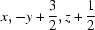; (iii) 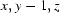; (iv) 
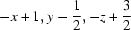
.

**Fig. 1.**
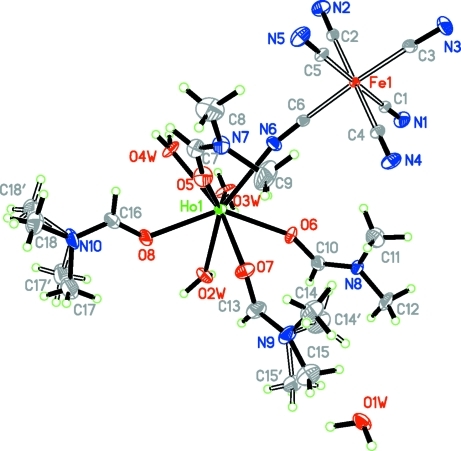
View of the molecule of complex (1) with atom-numbering scheme. Displacement ellipsoids are draw at the 30% probability level. Both positionally disordered dimethyl groups are shown.

**Fig. 2.**
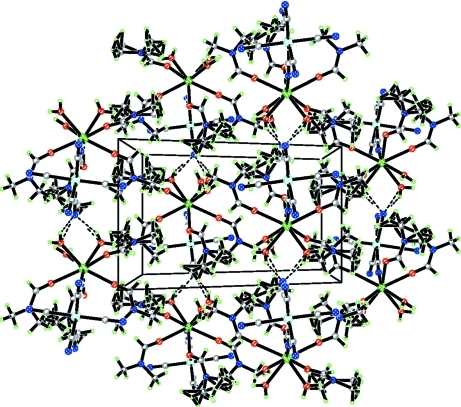
Packing diagram for complex (1) viewed down the c axis. Hydrogen bonds are shown as dashed lines.

## supplementary crystallographic information

### Comment

In 1998 Kou *et al.* obtained a binuclear Sm—Fe complex, [Sm Fe(DMF)_4_(H_2_O)_3_].H_2_O (DMF = *N, N*-dimethylformamide), using Sm(NO_3_)_3_ with DMF acting as assistant ligand. Later, some researchers reported lighter and heavier bimetallic complex rare earth ion cyanides [*Ln*Fe(CN)_6_(DMF)_4_(H_2_O)_3_].H_2_O (*Ln* = La, Nd, Gd, Pr and Eu). It is interesting to note that the number of coordinating water molecules is found to be different in these complexes. When *Ln* = Sm and Pr (Kou *et al.,* 1998, Dai *et al.*, 2004), there are four coordinating water molecules in the complex; however when *Ln* = La, Ce, Nd, Gd and Eu (Kautz *et al.*, 2000; Mullica *et al.*, 2000, Li, Akitsu *et al.*, 2003, and Li, Guo *et al.*, 2003), three coordinating water molecules are found. In order to further illustrate the influence of lanthanide contraction on the composition and structure of such complexes, we synthesized a new binuclear complex [HoFe(CN)_6_(DMF)_4_(H_2_O)_3_].H_2_O (I), of which the crystal structure reported.

As shown in Fig. 1, the structure of (I)  consists of neutral bimetallic HoFe(CN)_6_(DMF)_4_(H_2_O)_3_ complexes and solvent water molecules. The Ho^III^ and Fe^III^ ions are bridged by a cyanide group to form a binuclear complex. The Ho^III^ is eight-coordinated with one N atom of the bridging cyanide ligand [Ho—N= 2.572 (0) Å], four O atoms of DMF molecules [Ho—O_DMF_= 2.412 (3) Å-2.460 (7) Å], with an average distance of 2.440 (5) Å, and three water molecules, for which the three Ho—O_water_ distances are in the range 2.479 (9) Å-2.489 (1) Å, with an average distance of 2.485 (1) Å. The coordination polyhedron can be described as a slightly distorted square-antiprism. A similar situation was found in in [Nd Fe(DMF)_4_(H_2_O)_3_].H_2_O (Li, Akitsu *et al.*, 2003). The Ho1—N6—C6 angle is 163.4 (1)°, deviating slightly from linearity, as was the case in its analog. A three-dimensional framework is formed through O—H···N and O—H···O hydrogen bonding interactions with O···O distance of 2.660 (1)Å and average O···N separations of 2.839 (3) Å (Fig. 2).

### Experimental

The title complex (1) was prepared by addition of Ho(NO_3_)_3_ (0.35 g, 1.0 mmol) solution in a solvent mix of 15 ml DMF/H_2_O (*v: v* = 1: 1) and one equivalent of anhydrous K_3_Fe(CN)_6_ (0.33 g, 1.0 mmol). The reaction mixture was filtered and yellow single crystals suitable for X-ray analysis were obtained by slow evaporation of the solvent after a week. Yield: 81%. IR spectra were recorded on a FTS-40 infrared spectrometer KBr: 3610, 3400 (broad band center), 2939, 2132, 1648, 1497, 1382, 1114, 675 cm^-1^.

### Refinement

H atoms were placed in calculated positions with C—H = 0.93 Å, and refined in a riding model with *U*_iso_(H) = 1.2 *U*_eq_ (C). The H atoms of the water molecules were located in a difference map and  their bond lengths were set to 0.86Å and afterwards  refined using a riding model with *U*_iso_ (H) = 1.5 *U*_eq_ (O).

Since the initial refinement gave a large positive residual density on the Fe site, we suspected a contamination of the Fe site with a heavier atom, such as Co or Ru. Indeed, an elemental analysis of the crystal gave an elemental mass ratio of Fe:Ru= 48.18:0.8230 which indicates thus trace amounts of ruthenium. The source of the problem was found to be the iron salt precursor K_3_Fe(CN)_6_, whose elemental analysis also gave trace amounts of Ru and in addition much tinier trace amounts of Co. It was decided to fix the site occupancy factors of Fe and Ru to 0.985 and 0.015, respectively, since constrained (sum fixed to 1.0) as well as free refinement of the Fe and Ru occupancies gave much too high ocuupancies - up to 25% - for Ru. This is not logical in view of the mean Fe(Ru)—C distance which is 1.940 Å in the title compound, compared to  1.929 Å in the Cambridge Structural Database for Fe—C and 2.023 Å for Ru—C  in a similar cyanide environment.

Complex (I) was refined with 120 restraints, especially for modelling the disorder in the two dimethyl groups.  Two alternative sites for C14 and C15 were refined to give occupancies of 0.44 (3) and 0.56 (3), respectively. A similar disorder was found for the dimethyl group C17-C18 with site occupancy factors of 0.44 (3) and 0.56 (3), respectively. Positionally disordered atoms were refined using distance restraints (DIFX, AFIX) and ADP restraints (SIMU, ISOR). The sub

### Crystal data

**Table:**

[Fe_0.98_HoRu_0.02_(CN)_6_(C_3_H_7_NO)_4_(H_2_O)_3_]·H_2_O	*Z* = 4
*M**_r_* = 742.25	*F*(000) = 1485.8
Monoclinic, *P*2_1_/*c*	*D*_x_ = 1.575 Mg m^−^^3^
Hall symbol: -P 2ybc	Mo *K*α radiation, λ = 0.71073 Å
*a* = 17.6587 (15) Å	θ = 2.3–27.7°
*b* = 8.9235 (8) Å	µ = 3.03 mm^−^^1^
*c* = 25.2750 (16) Å	*T* = 293 K
β = 128.208 (4)°	Block, yellow
*V* = 3129.5 (4) Å^3^	0.30 × 0.25 × 0.20 mm

### Data collection

**Table:**

Bruker APEXII CCD area-detector diffractometer	6392 independent reflections
Radiation source: fine-focus sealed tube	5217 reflections with *I* > 2σ(*I*)
graphite	*R*_int_ = 0.030
φ and ω scans	θ_max_ = 26.4°, θ_min_ = 1.5°
Absorption correction: multi-scan (*SADABS*; Sheldrick, 1996)	*h* = −22→21
*T*_min_ = 0.464, *T*_max_ = 0.583	*k* = −9→11
19042 measured reflections	*l* = −25→31

### Refinement

**Table:**

Refinement on *F*^2^	Primary atom site location: structure-invariant direct methods
Least-squares matrix: full	Secondary atom site location: difference Fourier map
*R*[*F*^2^ > 2σ(*F*^2^)] = 0.042	Hydrogen site location: inferred from neighbouring sites
*wR*(*F*^2^) = 0.131	H-atom parameters constrained
*S* = 1.04	*w* = 1/[σ^2^(*F*_o_^2^) + (0.0763*P*)^2^ + 11.5358*P*] where *P* = (*F*_o_^2^ + 2*F*_c_^2^)/3
6392 reflections	(Δ/σ)_max_ = 0.001
393 parameters	Δρ_max_ = 1.95 e Å^−^^3^
120 restraints	Δρ_min_ = −1.04 e Å^−^^3^

### Special details

**Table:**

Geometry. All e.s.d.'s (except the e.s.d. in the dihedral angle between two l.s. planes) are estimated using the full covariance matrix. The cell e.s.d.'s are taken into account individually in the estimation of e.s.d.'s in distances, angles and torsion angles; correlations between e.s.d.'s in cell parameters are only used when they are defined by crystal symmetry. An approximate (isotropic) treatment of cell e.s.d.'s is used for estimating e.s.d.'s involving l.s. planes.
Refinement. Refinement of *F*^2^ against ALL reflections. The weighted *R*-factor *wR* and goodness of fit *S* are based on *F*^2^, conventional *R*-factors *R* are based on *F*, with *F* set to zero for negative *F*^2^. The threshold expression of *F*^2^ > σ(*F*^2^) is used only for calculating *R*-factors(gt) *etc*. and is not relevant to the choice of reflections for refinement. *R*-factors based on *F*^2^ are statistically about twice as large as those based on *F*, and *R*- factors based on ALL data will be even larger.

### Fractional atomic coordinates and isotropic or equivalent isotropic displacement parameters (Å^2^)

**Table:**

	*x*	*y*	*z*	*U*_iso_*/*U*_eq_	Occ. (<1)
Ho1	0.252155 (17)	−0.08435 (3)	0.728404 (12)	0.02581 (11)	
Ru1	0.25530 (4)	0.24860 (8)	0.54262 (3)	0.01431 (16)	0.02
Fe1	0.25530 (4)	0.24860 (8)	0.54262 (3)	0.01431 (16)	0.98
C1	0.3936 (4)	0.2162 (6)	0.5976 (3)	0.0230 (11)	
C2	0.2381 (4)	0.0899 (6)	0.4835 (3)	0.0230 (11)	
C3	0.2655 (4)	0.3958 (7)	0.4911 (3)	0.0288 (13)	
C4	0.2701 (4)	0.4092 (6)	0.6008 (3)	0.0242 (11)	
C5	0.1176 (4)	0.2763 (6)	0.4864 (3)	0.0249 (12)	
C6	0.2508 (4)	0.1071 (6)	0.5989 (3)	0.0223 (11)	
C7	0.0209 (5)	0.0851 (8)	0.5983 (4)	0.0399 (15)	
H7	−0.0257	0.0153	0.5685	0.048*	
C8	−0.0929 (6)	0.2763 (11)	0.5226 (4)	0.071 (3)	
H8A	−0.1302	0.1921	0.4947	0.107*	
H8B	−0.0827	0.3440	0.4981	0.107*	
H8C	−0.1270	0.3271	0.5357	0.107*	
C9	0.0751 (8)	0.3376 (11)	0.6257 (6)	0.088 (3)	
H9A	0.0517	0.4081	0.6412	0.132*	
H9B	0.0905	0.3892	0.6001	0.132*	
H9C	0.1320	0.2894	0.6637	0.132*	
C10	0.4879 (4)	0.0850 (7)	0.8340 (3)	0.0338 (14)	
H10	0.5275	0.0267	0.8725	0.041*	
C11	0.4612 (6)	0.3173 (10)	0.7772 (4)	0.060 (2)	
H11A	0.4301	0.2593	0.7366	0.090*	
H11B	0.5035	0.3893	0.7793	0.090*	
H11C	0.4134	0.3685	0.7771	0.090*	
C12	0.6071 (5)	0.2788 (9)	0.8950 (4)	0.052 (2)	
H12A	0.6377	0.2048	0.9300	0.079*	
H12B	0.5940	0.3667	0.9100	0.079*	
H12C	0.6489	0.3044	0.8842	0.079*	
C13	0.3206 (5)	0.1586 (9)	0.8541 (4)	0.0474 (18)	
H13	0.3481	0.0782	0.8839	0.057*	
C14	0.291 (2)	0.429 (3)	0.8260 (15)	0.062 (5)	0.44 (3)
H14A	0.3060	0.5220	0.8500	0.094*	0.44 (3)
H14B	0.2227	0.4119	0.7984	0.094*	0.44 (3)
H14C	0.3104	0.4351	0.7980	0.094*	0.44 (3)
C15	0.412 (2)	0.355 (4)	0.9386 (15)	0.070 (6)	0.44 (3)
H15A	0.4626	0.3972	0.9398	0.104*	0.44 (3)
H15B	0.4378	0.2818	0.9737	0.104*	0.44 (3)
H15C	0.3807	0.4330	0.9452	0.104*	0.44 (3)
C14'	0.335 (2)	0.412 (2)	0.8506 (13)	0.068 (5)	0.56 (3)
H14D	0.3357	0.3950	0.8135	0.103*	0.56 (3)
H14E	0.3848	0.4814	0.8815	0.103*	0.56 (3)
H14F	0.2734	0.4524	0.8342	0.103*	0.56 (3)
C15'	0.4194 (14)	0.296 (3)	0.9573 (9)	0.053 (4)	0.56 (3)
H15D	0.4818	0.3255	0.9717	0.079*	0.56 (3)
H15E	0.4237	0.1980	0.9749	0.079*	0.56 (3)
H15F	0.3972	0.3663	0.9735	0.079*	0.56 (3)
C16	0.0630 (5)	−0.2223 (10)	0.7166 (4)	0.0493 (19)	
H16	0.0279	−0.2026	0.6709	0.059*	
C17	0.0593 (17)	−0.268 (4)	0.8065 (11)	0.065 (5)	0.44 (3)
H17A	0.1265	−0.2451	0.8321	0.098*	0.44 (3)
H17B	0.0521	−0.3609	0.8224	0.098*	0.44 (3)
H17C	0.0274	−0.1895	0.8117	0.098*	0.44 (3)
C18	−0.0869 (17)	−0.287 (3)	0.6958 (14)	0.061 (5)	0.44 (3)
H18A	−0.1061	−0.2273	0.7172	0.092*	0.44 (3)
H18B	−0.1076	−0.3889	0.6921	0.092*	0.44 (3)
H18C	−0.1157	−0.2484	0.6517	0.092*	0.44 (3)
C17'	0.0698 (13)	−0.358 (3)	0.7984 (9)	0.069 (5)	0.56 (3)
H17D	0.1369	−0.3577	0.8182	0.103*	0.56 (3)
H17E	0.0475	−0.4590	0.7921	0.103*	0.56 (3)
H17F	0.0612	−0.3063	0.8278	0.103*	0.56 (3)
C18'	−0.0818 (13)	−0.347 (3)	0.6845 (10)	0.060 (4)	0.56 (3)
H18D	−0.1026	−0.3260	0.6400	0.089*	0.56 (3)
H18E	−0.1260	−0.3033	0.6901	0.089*	0.56 (3)
H18F	−0.0799	−0.4539	0.6905	0.089*	0.56 (3)
N1	0.4743 (3)	0.1966 (7)	0.6286 (3)	0.0383 (13)	
N2	0.2289 (4)	−0.0020 (7)	0.4488 (3)	0.0448 (14)	
N3	0.2758 (5)	0.4849 (7)	0.4632 (3)	0.0523 (16)	
N4	0.2771 (4)	0.5030 (6)	0.6335 (3)	0.0395 (13)	
N5	0.0349 (4)	0.2905 (7)	0.4522 (3)	0.0430 (14)	
N6	0.2497 (4)	0.0265 (6)	0.6334 (3)	0.0346 (12)	
N7	0.0003 (4)	0.2238 (6)	0.5829 (3)	0.0361 (12)	
N8	0.5167 (3)	0.2186 (5)	0.8351 (2)	0.0281 (11)	
N9	0.3484 (5)	0.2909 (8)	0.8801 (4)	0.0558 (18)	
N10	0.0164 (4)	−0.2836 (9)	0.7354 (3)	0.0557 (19)	
O5	0.0981 (3)	0.0361 (5)	0.6498 (2)	0.0430 (11)	
O6	0.4104 (3)	0.0281 (5)	0.7849 (2)	0.0395 (11)	
O7	0.2615 (4)	0.1286 (5)	0.7941 (3)	0.0431 (11)	
O8	0.1475 (3)	−0.1878 (6)	0.7525 (2)	0.0440 (12)	
O1W	0.3239 (4)	0.7435 (5)	0.9333 (2)	0.0412 (11)	
H1WA	0.3003	0.6695	0.9407	0.049*	
H1WB	0.3161	0.8209	0.9498	0.049*	
O2W	0.3524 (3)	−0.1900 (5)	0.8442 (2)	0.0350 (10)	
H2WA	0.3355	−0.2198	0.8680	0.042*	
H2WB	0.4025	−0.2419	0.8578	0.042*	
O3W	0.3578 (3)	−0.2810 (5)	0.7351 (2)	0.0393 (11)	
H3WA	0.3555	−0.3561	0.7129	0.047*	
H3WB	0.4132	−0.2862	0.7742	0.047*	
O4W	0.1595 (3)	−0.2847 (5)	0.6432 (2)	0.0348 (10)	
H4WB	0.1762	−0.3568	0.6298	0.042*	
H4WA	0.0984	−0.2742	0.6125	0.042*	

### Atomic displacement parameters (Å^2^)

**Table:**

	*U*^11^	*U*^22^	*U*^33^	*U*^12^	*U*^13^	*U*^23^
Ho1	0.02603 (16)	0.02548 (16)	0.02722 (16)	−0.00088 (10)	0.01712 (13)	−0.00049 (10)
Ru1	0.0126 (3)	0.0156 (3)	0.0124 (3)	−0.0006 (3)	0.0066 (3)	−0.0008 (3)
Fe1	0.0126 (3)	0.0156 (3)	0.0124 (3)	−0.0006 (3)	0.0066 (3)	−0.0008 (3)
C1	0.024 (3)	0.022 (3)	0.020 (3)	−0.004 (2)	0.013 (2)	−0.003 (2)
C2	0.018 (2)	0.025 (3)	0.021 (3)	0.000 (2)	0.009 (2)	0.001 (2)
C3	0.032 (3)	0.027 (3)	0.023 (3)	0.001 (2)	0.015 (3)	0.000 (2)
C4	0.015 (2)	0.028 (3)	0.022 (3)	0.001 (2)	0.008 (2)	−0.002 (2)
C5	0.021 (3)	0.025 (3)	0.024 (3)	−0.004 (2)	0.011 (2)	−0.007 (2)
C6	0.016 (2)	0.030 (3)	0.019 (2)	0.000 (2)	0.010 (2)	−0.003 (2)
C7	0.035 (3)	0.039 (4)	0.046 (4)	0.004 (3)	0.025 (3)	0.000 (3)
C8	0.054 (5)	0.079 (7)	0.053 (5)	0.032 (5)	0.020 (4)	0.029 (5)
C9	0.082 (7)	0.041 (5)	0.097 (8)	−0.013 (5)	0.033 (6)	−0.003 (5)
C10	0.031 (3)	0.037 (4)	0.035 (3)	−0.003 (3)	0.021 (3)	0.001 (3)
C11	0.065 (5)	0.048 (5)	0.049 (5)	0.003 (4)	0.027 (4)	0.017 (4)
C12	0.043 (4)	0.048 (5)	0.045 (4)	−0.025 (3)	0.016 (3)	−0.016 (3)
C13	0.044 (4)	0.049 (5)	0.052 (4)	−0.001 (3)	0.031 (4)	−0.020 (4)
C14	0.061 (8)	0.045 (7)	0.063 (8)	0.000 (6)	0.029 (6)	−0.010 (6)
C15	0.065 (7)	0.063 (8)	0.067 (8)	0.002 (7)	0.035 (6)	−0.013 (7)
C14'	0.068 (8)	0.057 (7)	0.066 (7)	−0.002 (6)	0.034 (6)	−0.012 (6)
C15'	0.057 (6)	0.052 (7)	0.047 (6)	−0.009 (5)	0.031 (5)	−0.014 (5)
C16	0.048 (4)	0.072 (6)	0.037 (4)	−0.011 (4)	0.031 (3)	0.002 (4)
C17	0.061 (7)	0.081 (9)	0.062 (7)	−0.007 (6)	0.042 (5)	0.010 (6)
C18	0.042 (6)	0.067 (8)	0.068 (7)	−0.005 (6)	0.031 (5)	0.001 (7)
C17'	0.060 (6)	0.077 (8)	0.064 (7)	−0.014 (6)	0.036 (5)	0.016 (6)
C18'	0.043 (6)	0.069 (8)	0.064 (7)	−0.010 (6)	0.032 (5)	0.002 (6)
N1	0.018 (2)	0.048 (3)	0.034 (3)	0.000 (2)	0.009 (2)	0.000 (2)
N2	0.059 (4)	0.037 (3)	0.045 (3)	−0.004 (3)	0.035 (3)	−0.017 (3)
N3	0.078 (5)	0.035 (3)	0.050 (4)	−0.010 (3)	0.043 (4)	0.007 (3)
N4	0.041 (3)	0.036 (3)	0.038 (3)	−0.001 (2)	0.023 (3)	−0.017 (3)
N5	0.020 (3)	0.046 (4)	0.045 (3)	0.004 (2)	0.011 (2)	−0.006 (3)
N6	0.042 (3)	0.036 (3)	0.031 (3)	0.001 (2)	0.025 (2)	0.007 (2)
N7	0.030 (3)	0.034 (3)	0.037 (3)	0.007 (2)	0.017 (2)	0.010 (2)
N8	0.024 (2)	0.026 (3)	0.028 (2)	−0.0027 (19)	0.013 (2)	0.000 (2)
N9	0.060 (4)	0.055 (4)	0.072 (4)	−0.026 (3)	0.050 (4)	−0.042 (4)
N10	0.026 (3)	0.095 (6)	0.045 (3)	−0.006 (3)	0.022 (3)	0.024 (3)
O5	0.032 (2)	0.038 (3)	0.050 (3)	0.016 (2)	0.021 (2)	0.014 (2)
O6	0.028 (2)	0.042 (3)	0.040 (3)	−0.019 (2)	0.017 (2)	−0.011 (2)
O7	0.053 (3)	0.032 (2)	0.044 (3)	−0.001 (2)	0.029 (2)	−0.015 (2)
O8	0.029 (2)	0.073 (4)	0.038 (3)	−0.011 (2)	0.025 (2)	0.001 (2)
O1W	0.065 (3)	0.034 (2)	0.052 (3)	0.002 (2)	0.049 (3)	0.001 (2)
O2W	0.030 (2)	0.051 (3)	0.033 (2)	0.0144 (19)	0.0234 (19)	0.017 (2)
O3W	0.0211 (19)	0.045 (3)	0.027 (2)	0.0093 (18)	0.0023 (17)	−0.0182 (19)
O4W	0.0138 (17)	0.038 (3)	0.037 (2)	−0.0037 (16)	0.0077 (17)	−0.0210 (19)

### Geometric parameters (Å, °)

**Table:**

Ho1—O5	2.412 (4)	C13—H13	0.9300
Ho1—O6	2.433 (4)	C14—N9	1.65 (3)
Ho1—O8	2.457 (4)	C14—H14A	0.9600
Ho1—O7	2.461 (4)	C14—H14B	0.9600
Ho1—O4W	2.480 (4)	C14—H14C	0.9600
Ho1—O2W	2.486 (4)	C15—N9	1.31 (3)
Ho1—O3W	2.489 (4)	C15—H15A	0.9600
Ho1—N6	2.572 (5)	C15—H15B	0.9600
Fe1—C5	1.929 (5)	C15—H15C	0.9600
Fe1—C3	1.935 (6)	C14'—N9	1.25 (2)
Fe1—C6	1.940 (6)	C14'—H14D	0.9600
Fe1—C2	1.941 (6)	C14'—H14E	0.9600
Fe1—C1	1.944 (5)	C14'—H14F	0.9600
Fe1—C4	1.951 (6)	C15'—N9	1.534 (19)
C1—N1	1.138 (7)	C15'—H15D	0.9600
C2—N2	1.138 (8)	C15'—H15E	0.9600
C3—N3	1.150 (8)	C15'—H15F	0.9600
C4—N4	1.130 (7)	C16—O8	1.213 (8)
C5—N5	1.155 (7)	C16—N10	1.297 (9)
C6—N6	1.140 (7)	C16—H16	0.9300
C7—O5	1.245 (8)	C17—N10	1.46 (2)
C7—N7	1.281 (8)	C17—H17A	0.9600
C7—H7	0.9300	C17—H17B	0.9600
C8—N7	1.465 (9)	C17—H17C	0.9600
C8—H8A	0.9600	C18—N10	1.44 (2)
C8—H8B	0.9600	C18—H18A	0.9600
C8—H8C	0.9600	C18—H18B	0.9600
C9—N7	1.476 (10)	C18—H18C	0.9600
C9—H9A	0.9600	C17'—N10	1.416 (18)
C9—H9B	0.9600	C17'—H17D	0.9600
C9—H9C	0.9600	C17'—H17E	0.9600
C10—O6	1.253 (8)	C17'—H17F	0.9600
C10—N8	1.289 (8)	C18'—N10	1.492 (19)
C10—H10	0.9300	C18'—H18D	0.9600
C11—N8	1.451 (9)	C18'—H18E	0.9600
C11—H11A	0.9600	C18'—H18F	0.9600
C11—H11B	0.9600	O1W—H1WA	0.8600
C11—H11C	0.9600	O1W—H1WB	0.8599
C12—N8	1.463 (8)	O2W—H2WA	0.8599
C12—H12A	0.9600	O2W—H2WB	0.8599
C12—H12B	0.9600	O3W—H3WA	0.8599
C12—H12C	0.9600	O3W—H3WB	0.8599
C13—O7	1.226 (9)	O4W—H4WB	0.8599
C13—N9	1.291 (9)	O4W—H4WA	0.8600
			
O5—Ho1—O6	126.91 (18)	N9—C13—H13	116.8
O5—Ho1—O8	74.53 (17)	N9—C14—H14A	109.5
O6—Ho1—O8	140.97 (15)	N9—C14—H14B	109.5
O5—Ho1—O7	77.44 (17)	N9—C14—H14C	109.5
O6—Ho1—O7	73.26 (16)	N9—C15—H15A	109.5
O8—Ho1—O7	82.50 (18)	N9—C15—H15B	109.5
O5—Ho1—O4W	78.68 (16)	N9—C15—H15C	109.5
O6—Ho1—O4W	135.11 (15)	N9—C14'—H14D	109.5
O8—Ho1—O4W	75.57 (16)	N9—C14'—H14E	109.5
O7—Ho1—O4W	151.08 (15)	H14D—C14'—H14E	109.5
O5—Ho1—O2W	139.24 (15)	N9—C14'—H14F	109.5
O6—Ho1—O2W	75.08 (15)	H14D—C14'—H14F	109.5
O8—Ho1—O2W	70.26 (14)	H14E—C14'—H14F	109.5
O7—Ho1—O2W	78.24 (16)	N9—C15'—H15D	109.5
O4W—Ho1—O2W	110.85 (15)	N9—C15'—H15E	109.5
O5—Ho1—O3W	141.85 (15)	H15D—C15'—H15E	109.5
O6—Ho1—O3W	73.17 (16)	N9—C15'—H15F	109.5
O8—Ho1—O3W	111.42 (17)	H15D—C15'—H15F	109.5
O7—Ho1—O3W	139.77 (15)	H15E—C15'—H15F	109.5
O4W—Ho1—O3W	67.38 (13)	O8—C16—N10	126.8 (7)
O2W—Ho1—O3W	72.22 (14)	O8—C16—H16	116.6
O5—Ho1—N6	72.59 (17)	N10—C16—H16	116.6
O6—Ho1—N6	74.89 (17)	N10—C17—H17A	109.5
O8—Ho1—N6	142.69 (16)	N10—C17—H17B	109.5
O7—Ho1—N6	106.76 (18)	N10—C17—H17C	109.5
O4W—Ho1—N6	81.13 (17)	N10—C18—H18A	109.5
O2W—Ho1—N6	146.47 (15)	N10—C18—H18B	109.5
O3W—Ho1—N6	85.08 (17)	N10—C18—H18C	109.5
C5—Ru1—C3	91.0 (3)	N10—C17'—H17D	109.5
C5—Ru1—C6	91.1 (2)	N10—C17'—H17E	109.5
C3—Ru1—C6	176.7 (2)	H17D—C17'—H17E	109.5
C5—Ru1—C2	90.0 (2)	N10—C17'—H17F	109.5
C3—Ru1—C2	90.7 (2)	H17D—C17'—H17F	109.5
C6—Ru1—C2	91.8 (2)	H17E—C17'—H17F	109.5
C5—Ru1—C1	178.4 (2)	N10—C18'—H18D	109.5
C3—Ru1—C1	89.1 (2)	N10—C18'—H18E	109.5
C6—Ru1—C1	89.0 (2)	H18D—C18'—H18E	109.5
C2—Ru1—C1	88.3 (2)	N10—C18'—H18F	109.5
C5—Ru1—C4	89.0 (2)	H18D—C18'—H18F	109.5
C3—Ru1—C4	89.0 (3)	H18E—C18'—H18F	109.5
C6—Ru1—C4	88.4 (2)	C6—N6—Ho1	163.4 (5)
C2—Ru1—C4	178.9 (2)	C7—N7—C8	123.3 (7)
C1—Ru1—C4	92.7 (2)	C7—N7—C9	119.0 (7)
N1—C1—Ru1	178.6 (5)	C8—N7—C9	117.6 (7)
N2—C2—Ru1	179.0 (6)	C10—N8—C11	121.9 (6)
N3—C3—Ru1	176.8 (6)	C10—N8—C12	121.7 (6)
N4—C4—Ru1	178.8 (6)	C11—N8—C12	116.4 (6)
N5—C5—Ru1	178.8 (6)	C14'—N9—C13	128.0 (12)
N6—C6—Ru1	178.2 (5)	C14'—N9—C15	90.6 (17)
O5—C7—N7	125.3 (7)	C13—N9—C15	139.8 (19)
O5—C7—H7	117.3	C14'—N9—C15'	116.1 (12)
N7—C7—H7	117.3	C13—N9—C15'	115.2 (11)
N7—C8—H8A	109.5	C15—N9—C15'	25.5 (15)
N7—C8—H8B	109.5	C14'—N9—C14	20.4 (13)
H8A—C8—H8B	109.5	C13—N9—C14	114.8 (11)
N7—C8—H8C	109.5	C15—N9—C14	105.5 (17)
H8A—C8—H8C	109.5	C15'—N9—C14	129.4 (12)
H8B—C8—H8C	109.5	C16—N10—C17'	118.5 (9)
N7—C9—H9A	109.5	C16—N10—C18	124.9 (13)
N7—C9—H9B	109.5	C17'—N10—C18	116.5 (14)
H9A—C9—H9B	109.5	C16—N10—C17	116.5 (11)
N7—C9—H9C	109.5	C17'—N10—C17	35.1 (11)
H9A—C9—H9C	109.5	C18—N10—C17	109.1 (17)
H9B—C9—H9C	109.5	C16—N10—C18'	120.5 (10)
O6—C10—N8	124.8 (6)	C17'—N10—C18'	113.7 (12)
O6—C10—H10	117.6	C18—N10—C18'	25.1 (11)
N8—C10—H10	117.6	C17—N10—C18'	122.5 (13)
N8—C11—H11A	109.5	C7—O5—Ho1	165.0 (5)
N8—C11—H11B	109.5	C10—O6—Ho1	154.5 (4)
H11A—C11—H11B	109.5	C13—O7—Ho1	130.9 (5)
N8—C11—H11C	109.5	C16—O8—Ho1	132.6 (4)
H11A—C11—H11C	109.5	H1WA—O1W—H1WB	105.6
H11B—C11—H11C	109.5	Ho1—O2W—H2WA	129.6
N8—C12—H12A	109.5	Ho1—O2W—H2WB	118.8
N8—C12—H12B	109.5	H2WA—O2W—H2WB	105.6
H12A—C12—H12B	109.5	Ho1—O3W—H3WA	140.8
N8—C12—H12C	109.5	Ho1—O3W—H3WB	112.9
H12A—C12—H12C	109.5	H3WA—O3W—H3WB	105.6
H12B—C12—H12C	109.5	Ho1—O4W—H4WB	132.7
O7—C13—N9	126.5 (9)	Ho1—O4W—H4WA	119.5
O7—C13—H13	116.8	H4WB—O4W—H4WA	105.6

### Hydrogen-bond geometry (Å, °)

**Table:**

*D*—H···*A*	*D*—H	H···*A*	*D*···*A*	*D*—H···*A*
O1W—H1WA···N2^i^	0.86	2.05	2.901 (7)	173
O1W—H1WB···N3^ii^	0.86	1.98	2.821 (7)	165
O2W—H2WA···O1W^iii^	0.86	1.82	2.660 (6)	167
O2W—H2WB···N1^iv^	0.86	2.06	2.875 (7)	159
O3W—H3WA···N4^iii^	0.86	2.02	2.795 (6)	149
O3W—H3WB···N1^iv^	0.86	1.99	2.839 (6)	168
O4W—H4WB···N4^iii^	0.86	2.13	2.931 (7)	155

Symmetry codes: (i) *x*, −*y*+1/2, *z*+1/2; (ii) *x*, −*y*+3/2, *z*+1/2; (iii) *x*, *y*−1, *z*; (iv) −*x*+1, *y*−1/2, −*z*+3/2.
